# Patient:innenzentrierte Versorgung – Anspruch und Umsetzung im deutschen Gesundheitswesen

**DOI:** 10.1007/s00103-025-04184-5

**Published:** 2026-01-28

**Authors:** Martin Härter, Isabelle Scholl, Uwe Koch-Gromus

**Affiliations:** Institut und Poliklinik für Medizinische Psychologie, Universitätsklinikum Hamburg-Eppendorf, Martinistr. 52, 20246 Hamburg, Deutschland

In den letzten Jahrzehnten hat sich das Gesundheitswesen zunehmend auf die Bedürfnisse und Perspektiven der Patient:innen ausgerichtet. Die Konzepte der Patient:innenautonomie, der Patient:innenzentrierung, aber auch der verbesserten Patient:innensteuerung sowie der Beschreibung von Patient Journeys haben international wie national erheblich an Bedeutung gewonnen. Das deutsche Gesundheitswesen, lange Zeit geprägt durch ein eher paternalistisches Modell, in dem der Arzt oder die Ärztin weitgehend alleine über Diagnostik und Therapie entschied, erlebte in den letzten Jahrzehnten einen fundamentalen Wandel. Die Entwicklung hin zu mehr Patient:innenzentrierung und partizipativer Entscheidungsfindung (PEF bzw. Shared Decision Making, SDM) hat sich von einem Leitbild zu einem wissenschaftlich fundierten und gesundheitspolitisch verankerten Qualitätsanspruch entwickelt und gilt heute als ethische und qualitative Notwendigkeit für eine hochwertige Versorgung. Die Bedeutung dieses Paradigmenwechsels ist enorm: Informierte und partizipative Entscheidungen fördern die Zufriedenheit von Patient:innen und Behandelnden, reduzieren Entscheidungskonflikte, erhöhen das Wissen über Behandlungen und die Patient:innensicherheit, um nur einige der wissenschaftlich belegten Effekte zu nennen.

Trotz dieser insgesamt positiven Entwicklung sind Patient:innenzentrierung und partizipative Entscheidungsfindung heute noch nicht flächendeckend als feste Grundsätze in der deutschen Gesundheitsversorgung verankert. Modellprojekte zeigen, dass die Implementierung erfolgreich sein kann und positive Effekte mit sich bringt. Die vollständige Integration von Patient:innenzentrierung und partizipativer Entscheidungsfindung erfordert kontinuierliche Anstrengungen auf allen Ebenen des Gesundheitswesens. Es ist ein Prozess, der von der Politik über die Ausbildung der unterschiedlichen Gesundheitsberufe, die Förderung der Selbsthilfe, die Gesundheitsbildung und Steigerung der Gesundheitskompetenz der Bürger:innen, die Qualitätssicherung, technologische Unterstützungssysteme bis hin zur individuellen Arzt-Patienten-Beziehung reicht.

Dieses Themenheft versammelt 11 wissenschaftliche Beiträge von Expert:innen aus dem Feld, die verschiedene Aspekte dieser wichtigen Themen beleuchten. Die ersten 3 Beiträge behandeln Grundlagen des Konzepts der Patient:innenzentrierung. Im ersten Beitrag berichten *Schröer-Günther et al., *wie das Institut für Qualität und Wirtschaftlichkeit im Gesundheitswesen (IQWiG) als Teilverpflichtung seines gesetzlichen Auftrags die Informationsprozesse für erkrankte und gesunde Bürger:innen gestaltet. Die Autor:innen führen den Erfolg ihrer Bemühungen auf gezielte Strategien zur Einbindung von Betroffenen und Patientenorganisationen zurück. *K. Blatt und V. Andorfer *vom Institut für Qualitätssicherung und Transparenz im Gesundheitswesen (IQTIG) setzen sich mit Maßnahmen der Qualitätssicherung auseinander, die auch Betroffene effektiv und bedarfsorientiert einbinden. Eine wichtige Rolle kommt dabei Patient:innenbefragungen unter Einbeziehung des Konzepts der partizipativen Entscheidungsfindung und Befragungsansätzen mittels Patient-reported Experience Measures (PREMs) und Patient-reported Outcome Measures (PROMs) zu. Der dritte Beitrag von *M. Geraedts et al.* stellt die Patient:innensicherheit als ein vordringliches Kriterium einer gelungenen Gesundheitsversorgung in den Mittelpunkt. Zielsetzungen sind dabei die Vermeidung unerwünschter Ereignisse, Aktivitäten zur Gewährleistung einer sicheren Versorgungsumgebung und die Implementierung einer auf Patient:innen fokussierten Sicherheitskultur. Die Autor:innen sehen in Deutschland nach wie vor einen hohen Forschungs- und Entwicklungsbedarf für die Gewährleistung von Patient:innensicherheit.

Im zweiten Themenabschnitt werden in 4 Beiträgen methodische Aspekte und Projekte behandelt. Zunächst geben *E. Christalle et al.* einen Überblick über Methoden und Fragebögen zur erlebten Patient:innenzentrierung. Die hier besonders wichtigen PREMs ermöglichen eine standardisierte Messung von Patient:innenerfahrungen. Hier sehen die Autor:innen in Deutschland erhebliche Entwicklungsrückstände. Speziell vorgestellt wird der „Fragebogen zur erlebten Patient:innenorientierung“ (EPAT), der Patient:innenzentrierung erstmals in Deutschland theoretisch fundiert, generisch und in valider Form erfasst. Er ist mit seiner Lang- und Kurzversion sowohl in der ambulanten als auch stationären Versorgung nutzbringend einsetzbar. *F. Geiger et al.* setzen sich mit der Frage auseinander, warum Konzepte des Shared Decision Making (SDM) in Deutschland bisher allenfalls in Ansätzen umgesetzt wurden und welche Wirkkräfte des deutschen Gesundheitssystems für diesen Stillstand verantwortlich sind. Als Beispiel für eine gelungene Umsetzung wird das SHARE-TO-CARE-Programm an der Universitätsklinik Kiel beschrieben. Die Autor:innen geben Hinweise auf mögliche Weichenstellungen des Gesetzgebers für eine erfolgreiche und nachhaltige SDM-Umsetzung. *L. Harst et al.* berichten über Erfahrungen mit dem Konzept der Gesundheitslots:innen im Rahmen von Projekten aus dem Innovationsfonds. Gesundheitslots:innen sollen Betroffenen eine Orientierung im Rahmen eines immer komplexer werdenden Gesundheitssystems ermöglichen. In das Scoping-Review wurden 31 Lots:innenprojekte mit einem großen Spektrum von Zielgruppen eingeschlossen. Die Ergebnisse sind sehr heterogen und weisen nur zum Teil auf positive Effekte des Einsatzes von Gesundheitslots:innen hin. Die Autor:innen fordern daher eine Vereinheitlichung des Reportings zu derartigen Projekten, um die Wirksamkeit von Lots:innen in Zukunft besser verständlich zu machen. *M. Rose* setzt sich im Rahmen seines Beitrags mit Patient-reported Outcomes als Instrument einer patientenzentrierten Versorgung auseinander. Die in den letzten Jahren entwickelten PROMs haben inzwischen eine große Bedeutung für eine patient:innenzentrierte Beurteilung des Behandlungserfolges gewonnen. Inhaltlich zielen sie auf die Erfassung von Symptomen, Alltagsfunktionen und verschiedenen Aspekten der Lebensqualität und werden in zahlreichen klinischen Studien, in der Qualitätssicherung und als wirksame Screening-Instrumente eingesetzt, um eine patient:innenorientierte Versorgung sicherzustellen. Der Autor diskutiert auch die Voraussetzungen, um PROMs-Ansätze in der klinischen Versorgung künftig noch gezielter umzusetzen.

Im dritten Abschnitt zum Themenbereich „Einbezug von Patient:innen“ versammeln sich 4 Beiträge. *L. Oster et al. *verstehen Patient:innenzentrierung als Anspruch, Patient:innen systematisch einzubeziehen. Sie werden somit nicht als Forschungssubjekt verstanden, sondern tragen als Expert:innen mit Krankheits- und Versorgungserfahrungen zum Forschungsprozess bei. Die Autor:innen beschreiben, wie in Deutschland derzeit die Umsetzung von Patient:innenbeteiligung durch Forschungsförderer und Patient:innenorganisationen erfolgreich forciert wird, verweisen aber auch darauf, dass die Umsetzung des Konzepts entsprechende Strukturen und Umsetzungswissen voraussetzt. Gleichzeitig mahnen sie eine kritische Reflexion an, wie Patient:innen sinnvoll und erfolgreich an Forschungsprojekten mitwirken können. Auch *M. Rams et al.* sehen Patient:innenautonomie als zentrales ethisches und gesundheitspolitisches Leitprinzip moderner Gesundheitsversorgung. Dieses Prinzip gewinnt vor allem vor dem Hintergrund einer rasch fortschreitenden Digitalisierung zunehmend an Bedeutung. Digitale Technologien verändern allerdings auch die Bedingungen, unter denen Autonomie ausgeübt werden kann. Die Berücksichtigung eines erweiterten Autonomieverständnisses stellt hohe Anforderungen an digitale Infrastrukturen. *C. Kofahl et al. *fokussieren die Rolle der Selbsthilfe-orientierten Forschung bei dem Bestreben, Patient:innenerfahrungen in die Patient:innenversorgung zu integrieren. Sie illustrieren dies am Beispiel der Krebsselbsthilfe und zeigen, dass Durchführbarkeit, Qualität und patient:innenseitige Relevanz onkologischer Studien durch die Berücksichtigung von Selbsthilfegruppen (SHG) und Selbsthilfeorganisationen (SHO) erheblich verbessert werden können. Die Autor:innen diskutieren erfolgsfördernde Rahmenbedingungen für eine gelingende Kooperation zwischen Wissenschaft und Selbsthilfe. Im abschließenden Beitrag analysieren S*. Khan-Gökkaya et al.* Barrieren und Diskriminierungserfahrungen von Patient:innen bei der Nutzung von Angeboten des Gesundheitswesens. Diskriminierung wird dabei als „Benachteiligung von Menschen ohne sachliche Rechtfertigung anhand ihrer (vermeintlichen) Zugehörigkeit zu einer sozial konstruierten Kategorie von Personen“ verstanden. Sie kann interpersonal oder institutionell erfolgen. Grundlagen von Diskriminierung sind besonders häufig Geschlecht und sexuelle Orientierung, migrationsbezogene und rassistische Barrieren, bestimmte Behinderungen oder (Vor‑)Erkrankungen sowie Armut oder soziale Ungleichheit. Im Beitrag werden auf der Grundlage der internationalen Literatur Ansätze beschrieben, die geeignet erscheinen, bei marginalisierten Gruppen Ansprüche der Patient:innenzentrierung besser umzusetzen.

Liebe Leser:innen, wir hoffen, dass es durch die Auswahl der Beiträge des Themenheftes gelungen ist, ein informatives Bild der aktuellen Forschung und Diskussion zum Thema „Patient:innenzentrierung“ zu vermitteln. Wir danken allen Autor:innen der Beiträge und den Mitarbeiterinnen der Redaktion des Bundesgesundheitsblattes für die sehr gute Zusammenarbeit. Ein besonderer Dank gilt den Patient:innenvertreter:innen, die als Co-Autor:innen aktiv an der Gestaltung des Heftes mitgewirkt haben und so dazu beitragen, dass Patient:innenzentrierung auch auf diesem Weg umgesetzt wird. Wir wünschen eine anregende Lektüre.

